# Re-evaluation of bovine herpesvirus 4 genotyping based on the thymidine kinase gene

**DOI:** 10.1128/jvi.00733-26

**Published:** 2026-06-22

**Authors:** Wentao Lu, Juan Pu, Jiali Luo, Shukai Zhang, Jie Cao, Chong Ma

**Affiliations:** 1 College of Veterinary Medicine, China Agricultural University 34752, Beijing, China; University of Toronto, Toronto, Ontario, Canada

**Keywords:** bovine herpesvirus 4, thymidine kinase (TK), ORF 20, systematic error, genotyping

## LETTER

**Fig 1:**
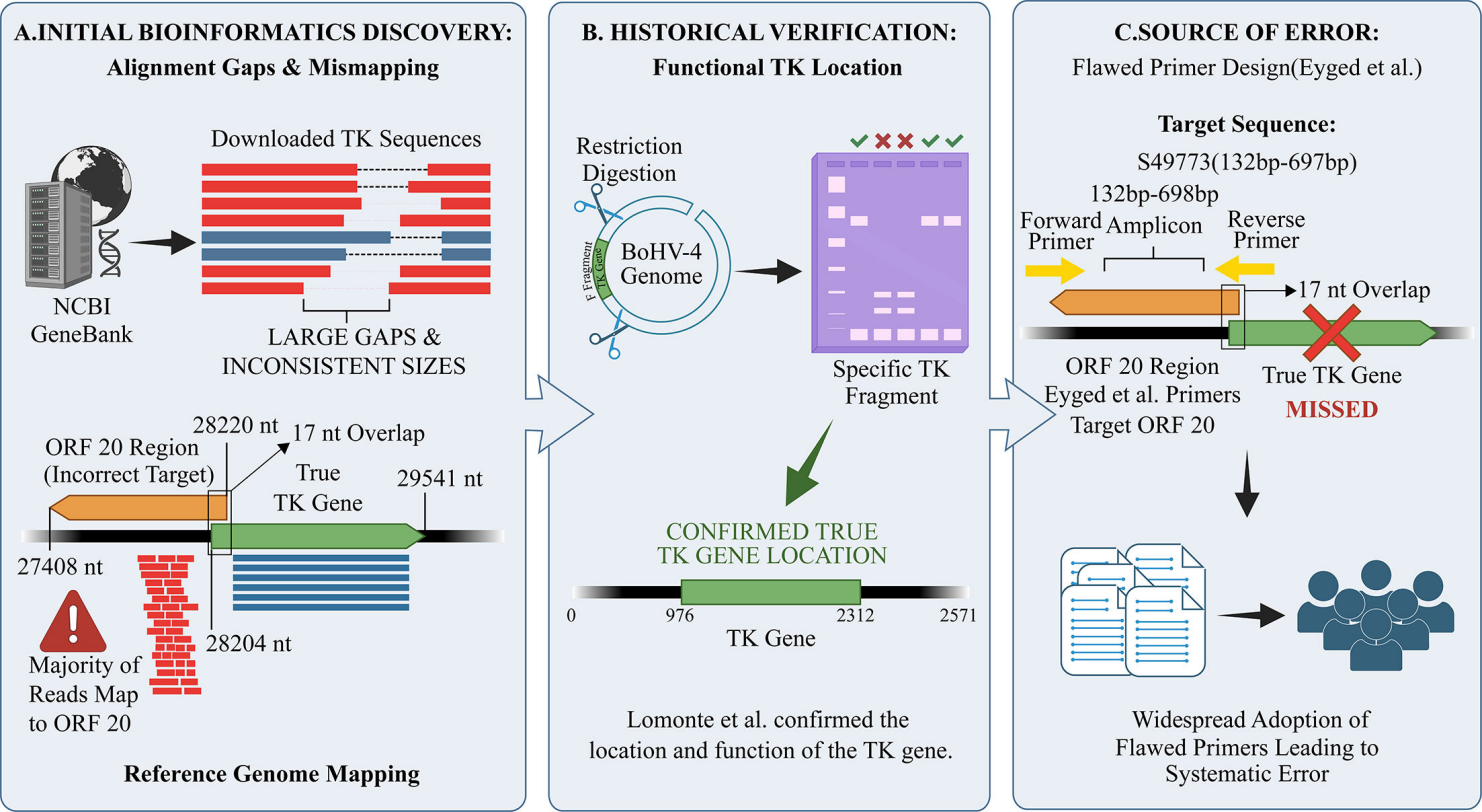
Identification and origin of systematic misannotation of the BoHV-4 thymidine kinase (TK) gene. (**A**) Initial bioinformatics discovery based on sequence alignment and reference genome mapping. (**B**) Historical verification of the functional TK gene location by restriction digestion. (**C**) Proposed source of error related to primer design and subsequent sequence annotation.

The thymidine kinase (TK) gene has been used as a core marker for bovine herpesvirus 4 (BoHV-4) genotyping (1). However, our re-evaluation of published data shows that the classical TK primers widely adopted in the field do not amplify the TK gene (2) (Table 1). Instead, they target ORF 20, leading to a long-standing and widespread misidentification in BoHV-4 molecular typing (Fig. 1).

**TABLE 1:** Primer sequences described by Egyed et al. (2) used in previous BoHV-4 studies

No.	Name	Sequence	Length
Eyged’s Primer 1	Eyged’s Primer F1	5′-GTTGGGCGTCCTGTATGGTAGC-3′	567 bp
	Eyged’s Primer R1	5′-ATGTATGCCCAAAACTTATAATATGACCAG-3′	
Eyged’s Primer 2	Eyged’s Primer F2	5′-TTGATAGTGCGTTGTTGGGATGTGG-3′	260 bp
	Eyged’s Primer R2	5′-CACTGCCCGGTGGGAAATAGCA-3′	

**Fig 2:**
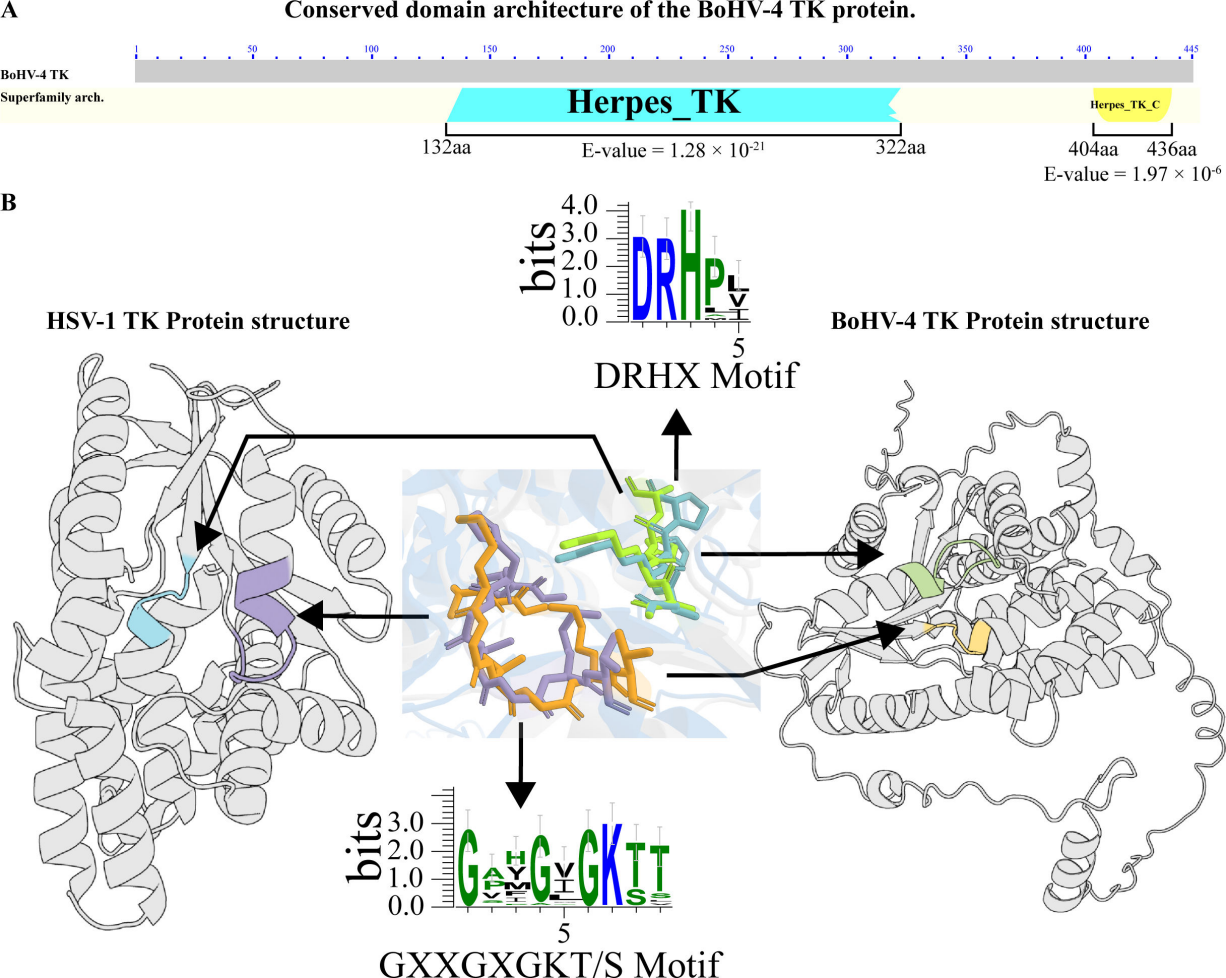
Conserved domain architecture and structural visualization of conserved motifs in the BoHV-4 TK protein. (**A**) Conserved domain analysis of the bona fide BoHV-4 TK protein using NCBI CD-Search/Pfam. (**B**) Structural visualization of conserved motifs in herpesvirus TK proteins (Pymol Version = 3.1.6.1).

**Fig 3:**
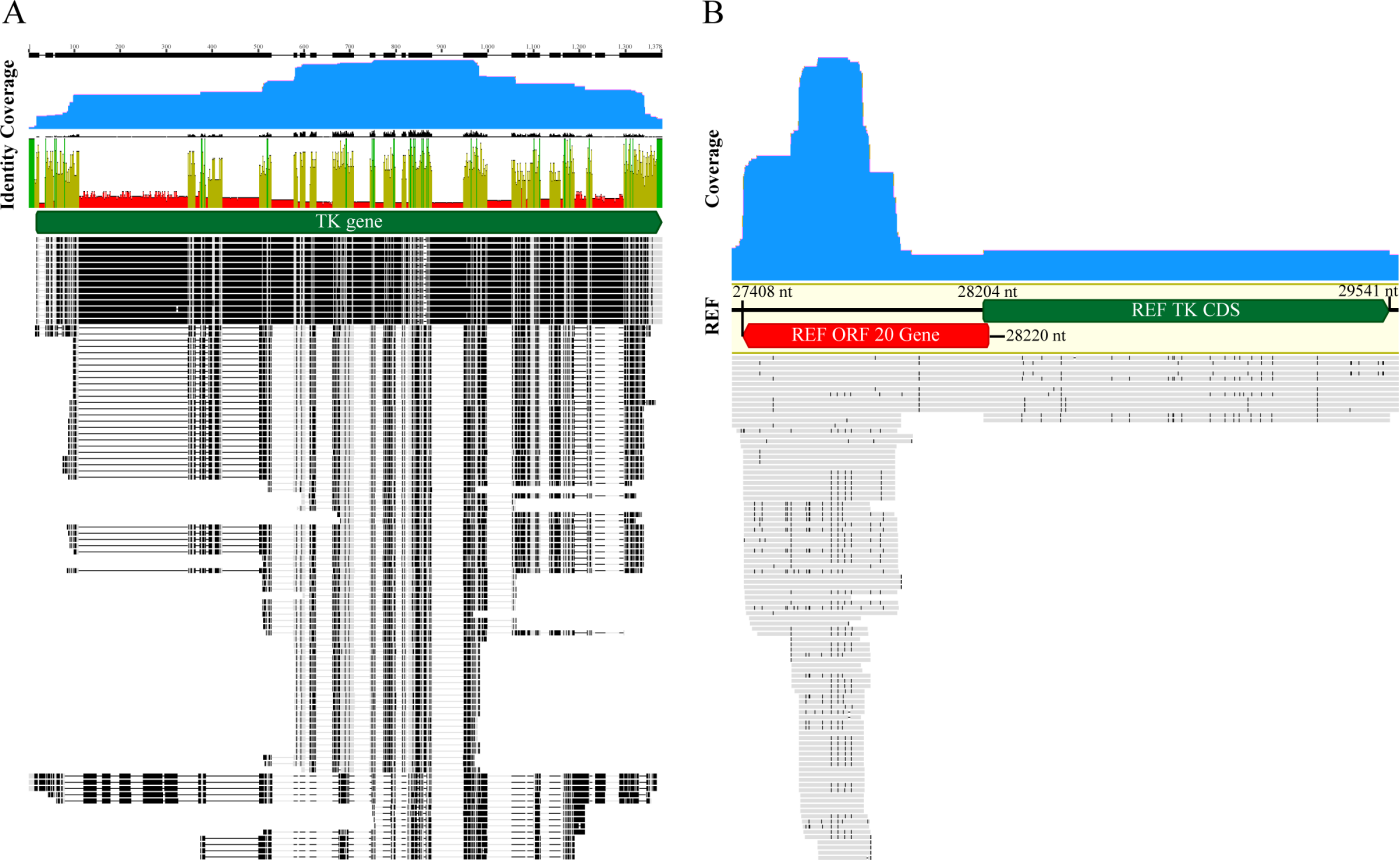
Multiple sequence alignment (**A**) and reference-based alignment (**B**) of sequences annotated as BoHV-4 thymidine kinase (TK) genes deposited in GenBank.

We systematically investigate BoHV-4 sequences annotated as the TK gene in GenBank (search query: txid10385 [Organism] AND ("Thymidine Kinase"[All Fields] OR TK[All Fields]), date: 14 Dec 2025). Among 100 retrieved sequences, including 13 complete genomes, only 14 mapped to the authentic TK gene region of the reference genome, whereas the vast majority mapped to ORF 20. Thus, approximately 84% of sequences deposited as TK are in fact ORF 20 fragments (Table S1). We then traced the historical basis of BoHV-4 TK gene detection. Lomonte et al. originally mapped the TK gene to the 2,571-bp EcoRI F fragment, where it begins at nucleotide 976 and spans 1,335 nt (GenBank Accession No: S49773) (3). In the BoHV-4 reference genome (NC_002665), ORF 20 (27,408–28,220) and the TK gene (28,204–29,541) are situated on the antisense and sense strands, respectively. These two genes exhibit a 17-bp overlap at their 5′-terminal coding regions. Importantly, that study also compared the BoHV-4 TK protein with TK homologs from other herpesviruses and identified conserved amino acid features supporting its identity as a bona fide TK. In the present study, we further identified Herpes_TK and Herpes_TK_C domains in the bona fide BoHV-4 TK protein using NCBI CD-Search/Pfam. In addition, multiple sequence alignment and structural comparison showed that BoHV-4 TK retains conserved motifs and amino acid residues corresponding to the ATP-binding/glycine-rich loop-like region and other conserved TK regions (Fig. 2; Fig. S1). However, the nested PCR primers later introduced by Egyed et al. were designed in the 132–698 bp region of that same fragment, outside the authentic TK gene (2). This positional mismatch explains why the classical assay amplified ORF 20-derived fragments that were subsequently treated as TK sequences. Consistent with this finding, our review of previous phylogenetic studies showed that most studies referred to these amplicons as TK-derived, although the underlying target was actually ORF 20 (Table 2; Fig. 3).

**TABLE 2:** Evidence for widespread ORF 20-based misannotation in BoHV-4 TK gene phylogenetic studies

No.	Reference	Stated phylogenetic target	Primer reference	Amplicon length (bp)^*a*^	Actual genomic locus	Re-evaluation
1	Isolation and complete genomic characterization of a Movar 33/63-like Japanese bovine herpesvirus 4 from a calf with respiratory disease (4)	ORF 20	WGS	–	ORF 20	Standard
2	Bovine herpes virus type-4 infection among postpartum dairy cows in California: risk factors and phylogenetic analysis (5)	TK	Egyed et al. (2)	531	ORF 20	Misidentified
3	Whole genome sequence-based analysis of bovine gammaherpesvirus 4 isolated from bovine abortions (6)	TK	WGS	–	ORF 20	Misidentified
4	Characterization and phylogenetic analysis of bovine gammaherpesvirus 4 isolated in China, 2022 (7)	TK	Designed in this study	–	ORF 20	Misidentified
5	Isolation and molecular characterization of bovine herpesvirus 4 from cattle in mainland China (8)	TK	Egyed et al. (2)	576	ORF 20	Misidentified
6	Genomic analysis of bovine herpesvirus type 4 (BoHV-4) from Argentina: High genetic variability and novel phylogenetic groups (9)	TK	Egyed et al. (2)	–	ORF 20	Misidentified
7	A phylogenetic and genotyping study of bovine herpesvirus type 4 (BHV-4) in Turkey (10)	TK	Egyed et al. (2)	–	ORF 20	Misidentified
8	Genetic variability of bovine herpesvirus type 4 (BoHV-4) field strains from Turkish cattle herds (11)	TK	Egyed et al. (2)	567	ORF 20	Misidentified
9	Molecular characterization of the first bovine herpesvirus 4 (BoHV-4) strains isolated from *in vitro* bovine embryos production in Argentina (12)	TK	Egyed et al. (2)	212	ORF 20	Misidentified
10	Studies on the molecular biological peculiarities of bovine herpesvirus 4 (13)	TK	Egyed et al. (2)	567	ORF 20	Misidentified
11	Molecular biological characteristics of bovine gamma herpesvirus 4 originated from different clinical sources (14)	TK	Egyed et al. (2)	567	ORF 20	Misidentified
12	Characterization of the first bovine gammaherpesvirus 4 strain isolated from an aborted bovine fetus in Argentina (15)	TK	Egyed et al. (2)	567	ORF 20	Misidentified

^*a*^ – indicates that the exact length of the amplified fragment was not specified in the original publication.

**Fig 4:**
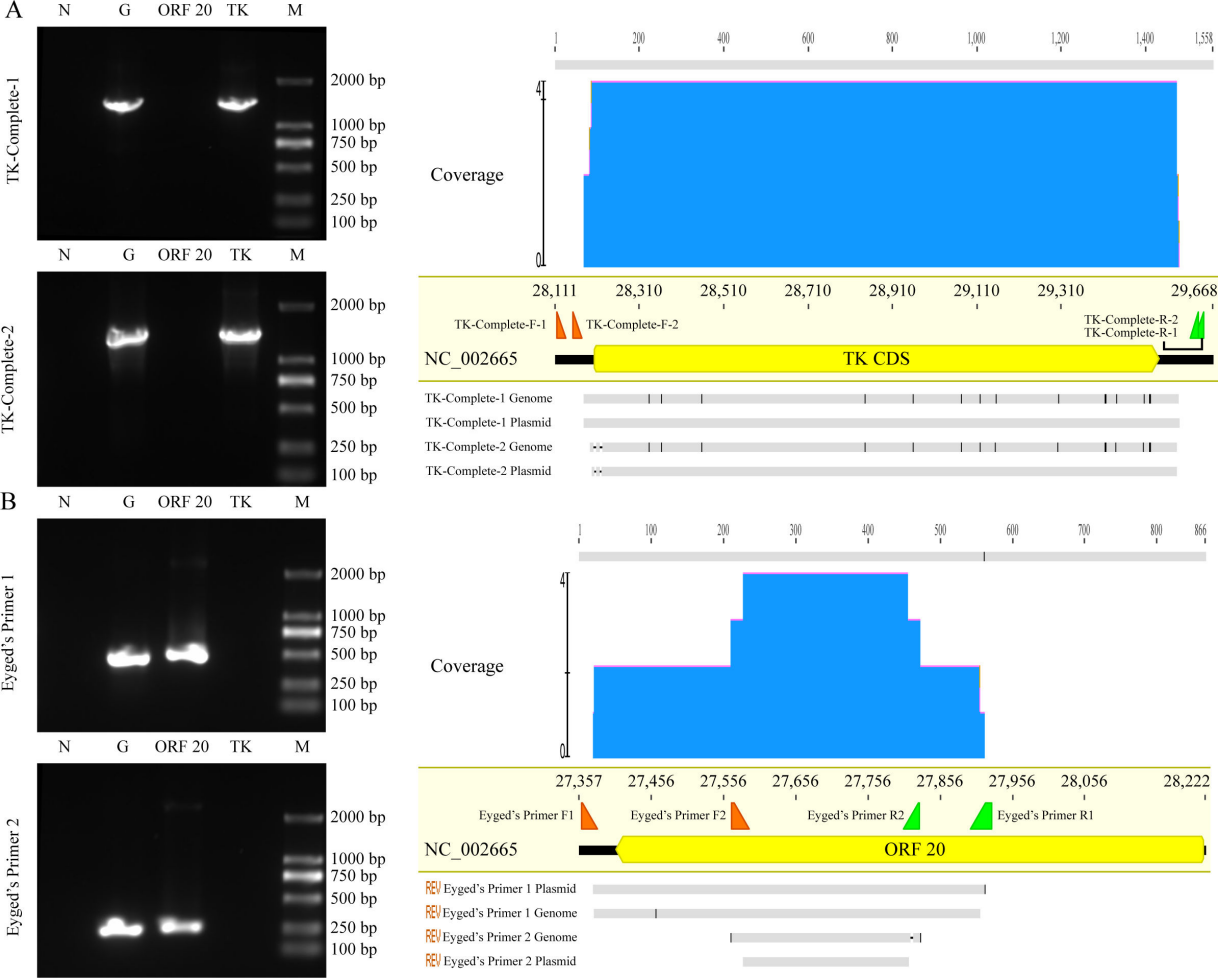
PCR amplification and sequencing coverage analyses reveal differences in primer specificity. (**A**) Specificity validation of the newly designed full-length TK primers (TK-Complete). (**B**) Re-evaluation of primer specificity reported in previous studies (Egyed et al. [2]). N, negative control; M, DNA marker; G, BoHV-4 genomic DNA; ORF 20, ORF 20-containing plasmid; TK, TK-containing plasmid.

We then experimentally tested the identity of the classical primers using BoHV-4 genomic DNA, an ORF 20 plasmid, and a plasmid containing the authentic full-length TK gene. The classical primers amplified the viral genome and the ORF 20 plasmid but failed to amplify the TK plasmid. Sequencing of the PCR products confirmed that the amplicons mapped entirely to ORF 20 and not to the TK coding region (Fig. 4B). These results demonstrate that the primers historically used for TK gene molecular typing in BoHV-4 do not target the TK gene at all.

**Fig 5:**
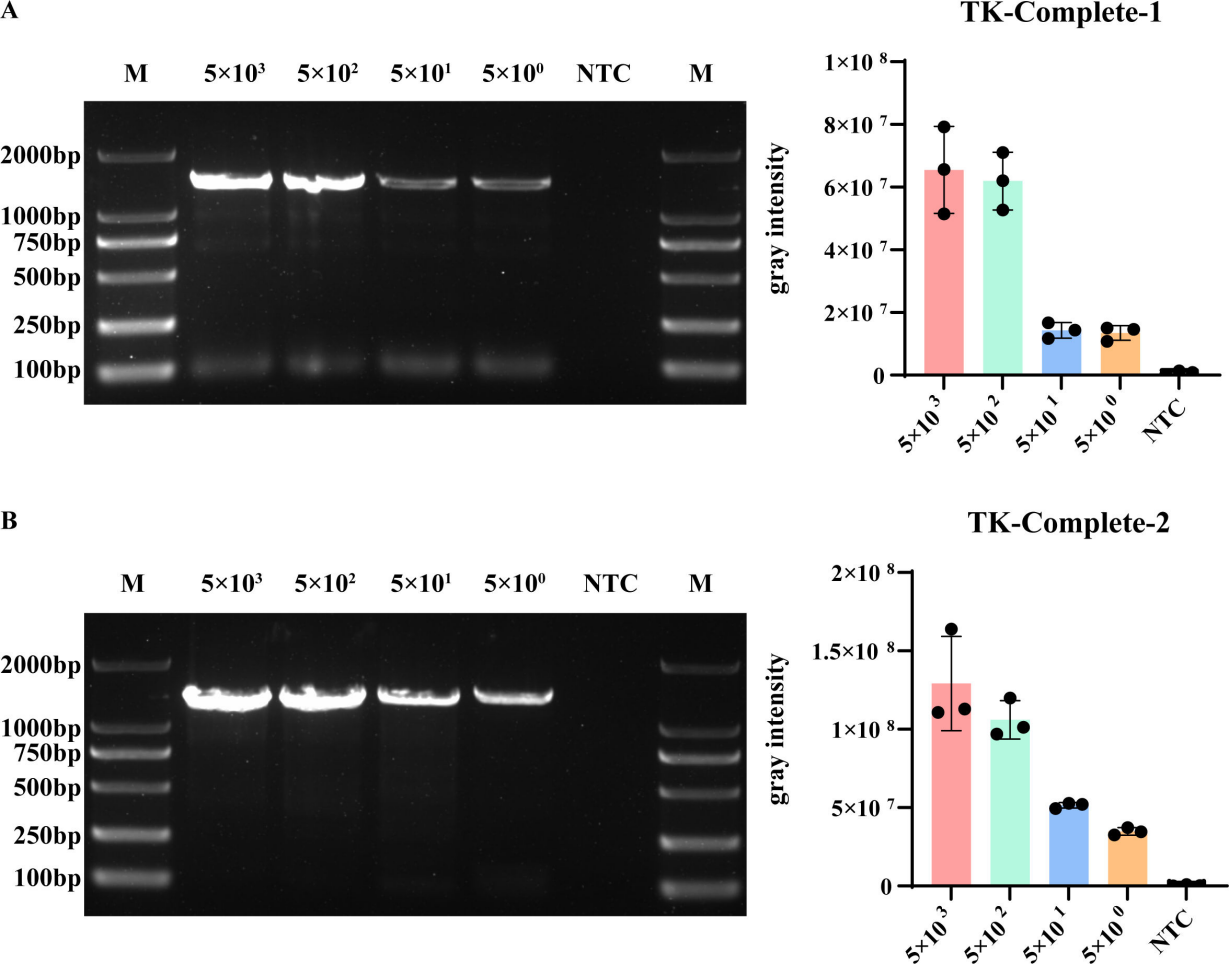
Analytical sensitivity of the newly designed full-length TK primers. (**A**) Agarose gel electrophoresis and densitometric analysis of TK-Complete-1 and (**B**) TK-Complete-2. Ten-fold serial dilutions of the BoHV-4 DNA standard, ranging from 5 × 10^3^ to 5 × 10°copies per reaction, were used as templates. Band intensities are presented as mean ± SD from three independent experiments (*n* = 3). M, DNA marker; NTC, no-template control.

To correct this problem, we designed new primer pairs spanning the authentic TK gene and validated them by PCR and Sanger sequencing (Table 3). These primers specifically amplified the full-length TK gene, producing the expected products from BoHV-4 genomic DNA and the TK plasmid, with no amplification from the ORF 20 plasmid. Sequence mapping confirmed correct coverage of the TK gene region (Fig. 4A). This provides a practical solution for future BoHV-4 genotyping based on the authentic TK gene. To further evaluate the performance of the newly designed TK primers, we assessed their analytical sensitivity using 10-fold serial dilutions of a BoHV-4 genomic DNA standard $(9.0\pm 1.8)\times 10^{6}$ copies/mL. The template copy number ranged from $5\times 10^{3}$ to $5\times 10^{0}$ copies per reaction. Both TK-Complete-1 and TK-Complete-2 amplified the expected target fragment over the range of $5\times 10^{3}$ to $5\times 10^{0}$ copies per reaction. Therefore, the detection limit of both primer sets was determined to be $5\times 10^{0}$ copies per reaction. Gray intensity analysis of the agarose gel bands showed that the target band intensity generally decreased with serial dilution of the template (Fig. 5).

**TABLE 3:** Primer pairs designed for amplification of the full-length TK gene

No.	Name	Sequence	Length
TK-Complete-1	TK-Complete-F-1	5′-GGTATCTAGGTGTGCCCTCTG-3′	1,532 bp
	TK-Complete-R-1	5′-CAGGTTTTCCACAAGACTCCC-3′	
TK-Complete-2	TK-Complete-F-2	5′-CCCAGTGGAAGGGTAGAGAGG-3′	1,483 bp
	TK-Complete-R-2	5′-AAGACTCCCCAAATCCCCC-3′	

**Fig 6:**
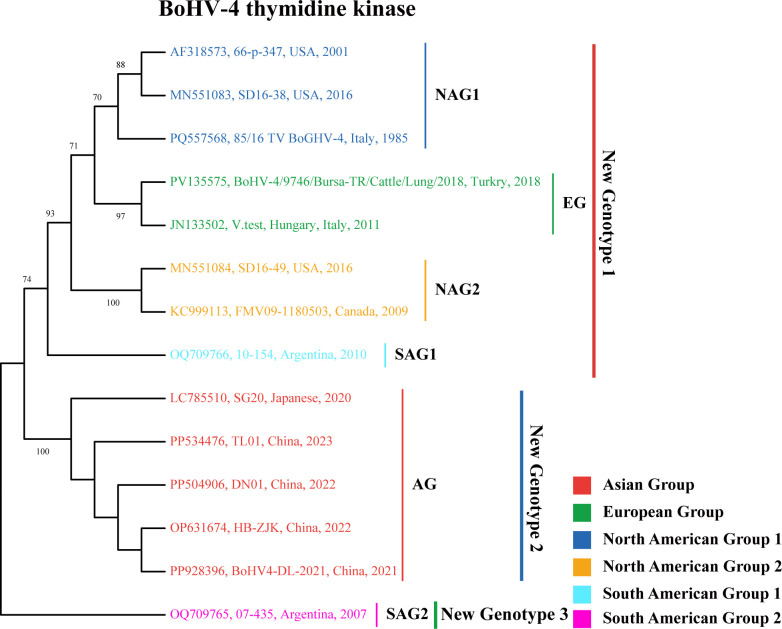
Comparative phylogenetic analysis of BoHV-4 based on the full-length TK gene. (The optimal nucleotide substitution model (T92 +I) was selected based on model testing performed in MEGA.)

Using complete TK sequences, we re-examined BoHV-4 phylogeny. In contrast to the historically used short ORF 20 fragments, which showed limited variation and poor phylogenetic resolution, the full-length TK gene revealed substantially greater diversity and a clearer geographic structure. Based on these data, BoHV-4 could be resolved into three major genotypes: New Genotype 1, comprising Euro-American strains; New Genotype 2, comprising Asia-specific strains; and New Genotype 3, represented by a distinct South American genotype. Within New Genotype 1, additional subdivision into North American, European, and South American groups was supported (Fig. 6).

Our study shows that the classical target used for BoHV-4 TK genotyping is ORF 20 rather than the TK gene region. The widespread use of ORF 20 in place of the TK gene has obscured the true evolutionary relationships of BoHV-4 and introduced substantial bias into previous classifications. By establishing validated full-length TK primers and reanalyzing BoHV-4 diversity on the basis of the authentic TK gene, this study provides a more reliable framework for future molecular epidemiology and phylogenetic analysis of BoHV-4.

## SUPPLEMENTAL MATERIAL

Fig. S1 jvi.00733-26-s0001.docx Multiple sequence alignment of BoHV-4 TK with TK proteins from 16 herpesviruses.

Table S1 jvi.00733-26-s0002.xlsx Summary of BoHV-4 TK gene sequences submitted to GenBank.

ASM does not own the copyrights to Supplemental Material that may be linked to, or accessed through, an article. The authors have granted ASM a non-exclusive, world-wide license to publish the Supplemental Material files. Please contact the corresponding author directly for reuse.
