# Supplementary material for: Re-evaluation of bovine herpesvirus 4 genotyping based on the thymidine kinase gene
Source: J Virol. 2026 Jun 22;100(7):e00733-26. doi: 10.1128/jvi.00733-26 (PMC13386966; doi:10.1128/jvi.00733-26)
Supplement: Fig. S1 — Multiple sequence alignment of BoHV-4 TK with TK proteins from 16 herpesviruses. [file jvi.00733-26-s0001.docx]

**Multiple sequence alignment of BoHV-4 TK with TK proteins from 16 herpesviruses**


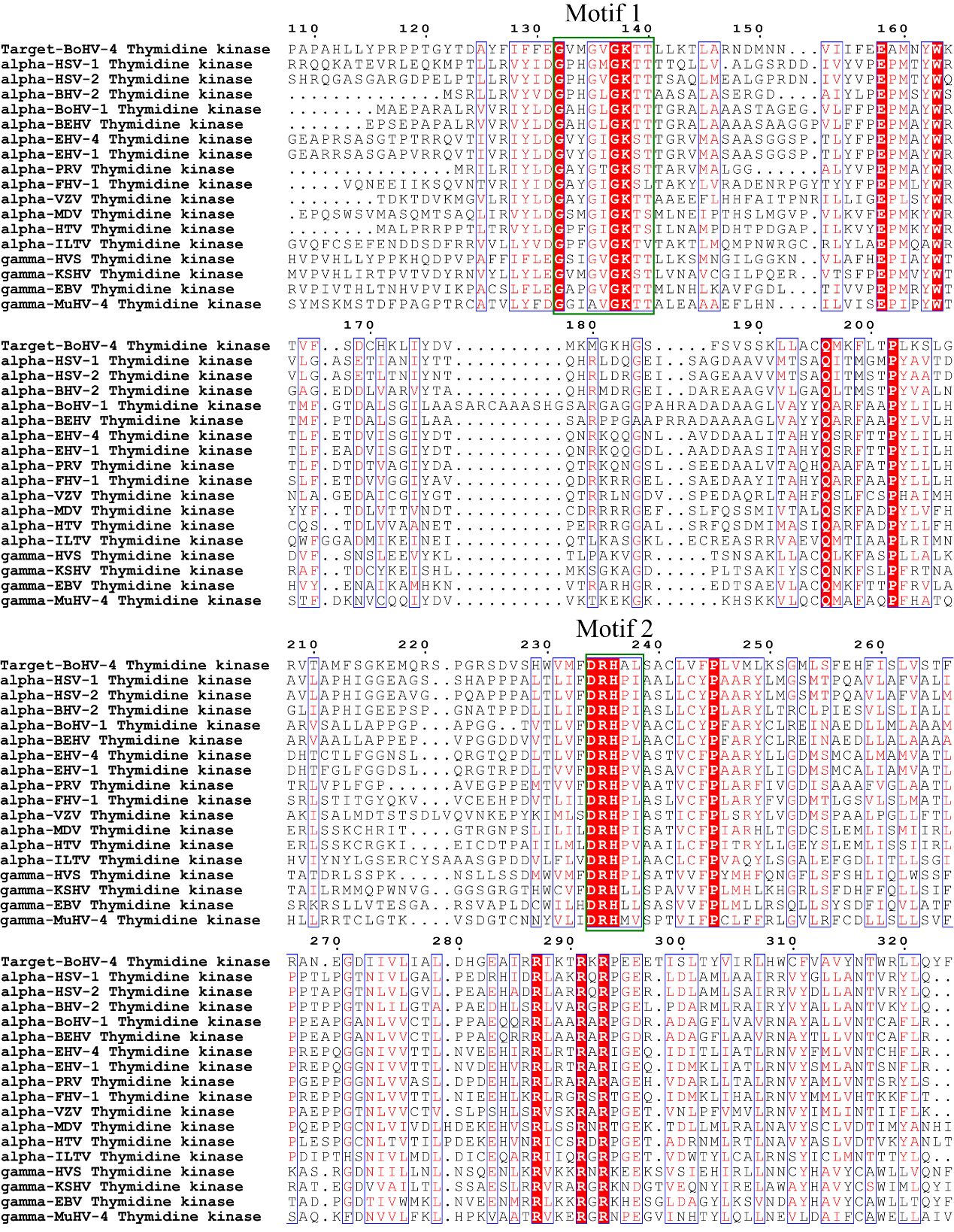

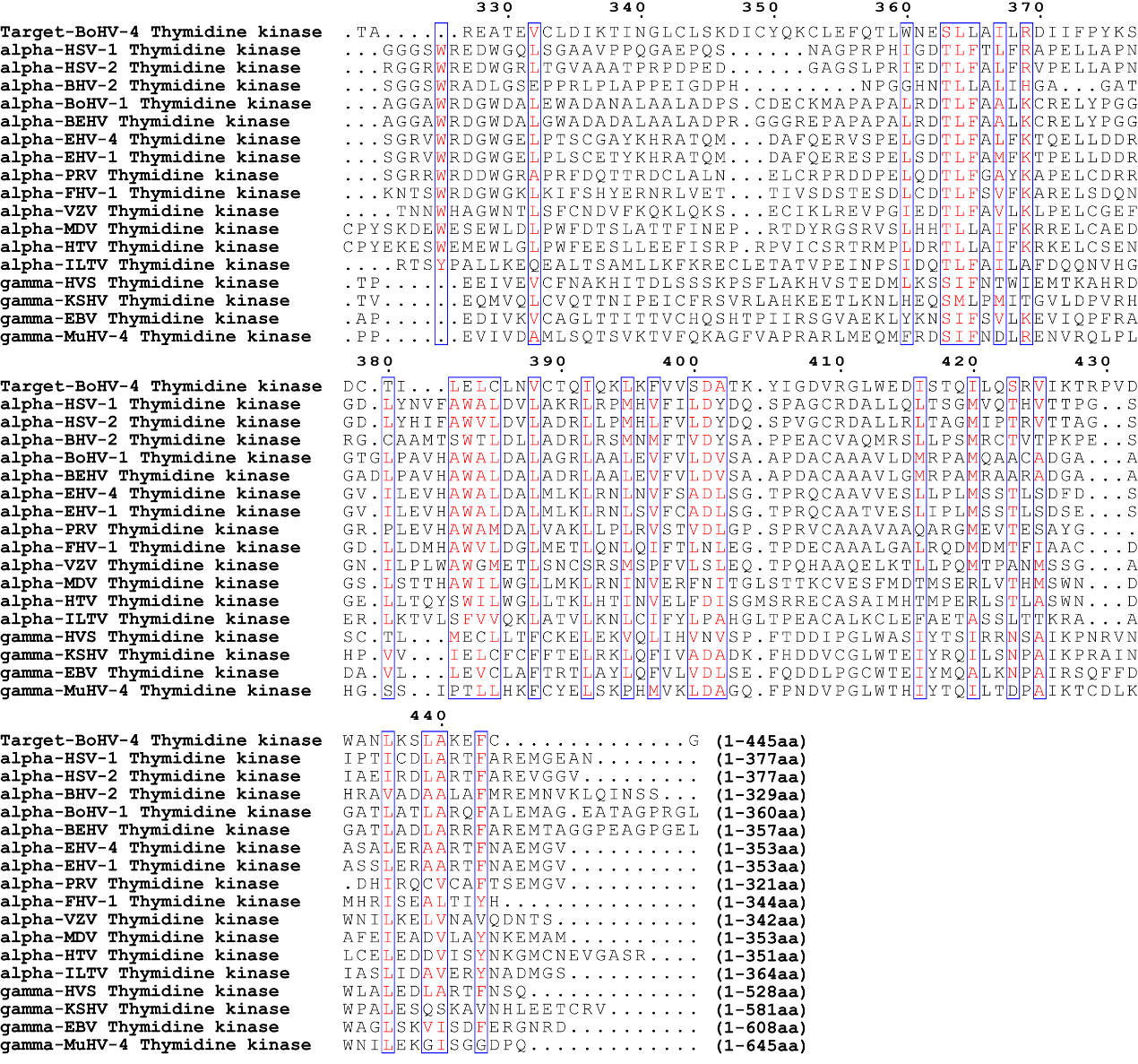


**Supplementary Fig. 1** Multiple sequence alignment of BoHV-4 TK with representative herpesvirus TK proteins. Multiple sequence alignment of the TK protein sequences was performed using MAFFT v7.490 within Geneious Prime. The resulting alignment was visualized and annotated using ESPript 3.2 to identify conserved domains and critical amino acid residues. Conserved residues are highlighted in color, while highly conserved Motifs are demarcated by green boxes.
